# Subcutaneous Leukemia Cutis as the Initial Manifestation of CD23 Negative CLL/SLL in a Patient With Rheumatoid Arthritis on Chronic TNF-Alpha Inhibition

**DOI:** 10.1155/crh/9950134

**Published:** 2025-08-21

**Authors:** Nehaal Ahmed, Saad Rashid, Nadeem Kutaish, Mohammed M. Ahmed

**Affiliations:** ^1^Division of Internal Medicine, Mayo Clinic, Rochester, Minnesota, USA; ^2^St. George's University School of Medicine, True Blue, Grenada; ^3^ProMedica Pathology Department, Consultants in Laboratory Medicine, Toledo, Ohio, USA; ^4^Department of Rheumatology, Arthritis & Rheumatism Center, Toledo, Ohio, USA

## Abstract

Rheumatoid arthritis (RA) is a chronic, systemic, and autoimmune disease characterized by inflammation and pain in the joints. While RA and TNF-alpha inhibitors have historically been associated with an increased risk of lymphoma, chronic lymphocytic leukemia/small lymphocytic lymphoma (CLL/SLL) is infrequently seen. CD23 negative CLL is rare. Extranodal manifestations of CLL/SLL are uncommon. While cutaneous involvement is among the more common extranodal manifestations, leukemia cutis is rare. Furthermore, subcutaneous leukemia cutis as the initial manifestation CLL/SLL is exceedingly uncommon. We describe a patient with longstanding RA on chronic TNF-alpha inhibition who presented with an isolated subcutaneous mass. Excisional biopsy demonstrated sheets of small, uniform, and mature lymphocytes with flow cytometric analysis noting a monoclonal B-cell population negative for CD23 expression but positive for CD5, CD19, CD20, CD38, kappa light chain, and CD200 expression. Further immunostaining was negative for cyclin-D1 and SOX11 and positive for CD43 and LEF1, overall consistent with CLL/SLL-induced subcutaneous leukemia cutis. While treatments for CLL/SLL-induced leukemia cutis vary, in this case, consolidative local radiation led to resolution of the remaining cutaneous lesion. Caution is advised when considering the use of TNF-alpha inhibitors in patients with a history of lymphoma.

## 1. Introduction

Chronic lymphocytic leukemia/small lymphocytic lymphoma (CLL/SLL) is an indolent B-cell lymphoma characterized by the accumulation of primarily small, mature leukemic cells in the peripheral blood, bone marrow, and lymphatic system [1–3]. CLL and SLL are different manifestations of the same disease. CLL is characterized by the abnormal lymphocytes primarily distributed in the peripheral blood, whereas in SLL, their primary residence is the bone marrow and lymphatic tissues [1]. CLL/SLL is the most prevalent adult leukemia in the West, with a median age at diagnosis of 71 [1, 2, 4]. Patients are generally asymptomatic at diagnosis, although common symptoms include fatigue, hepatosplenomegaly, lymphadenopathy, cytopenias secondary to bone marrow involvement or autoimmune destruction, sinopulmonary infections from hypogammaglobulinemia, and B symptoms, particularly in advanced disease [2–4].

While CLL/SLL is associated with various autoimmune conditions, it is infrequently seen with rheumatoid arthritis (RA) [5]. Additionally, it is uncommon for CLL/SLL to be CD23 negative and involve extranodal and extramedullary organs [2, 4]. Although cutaneous complications in CLL/SLL are well-reported, leukemia cutis (infiltration of the skin with leukemic cells) is rare [2, 4, 6]. We describe a case of a patient with longstanding seropositive RA who developed isolated subcutaneous leukemia cutis as the initial manifestation of CLL/SLL, an exceedingly rare phenomenon.

## 2. Case Summary

**Figure 1:**
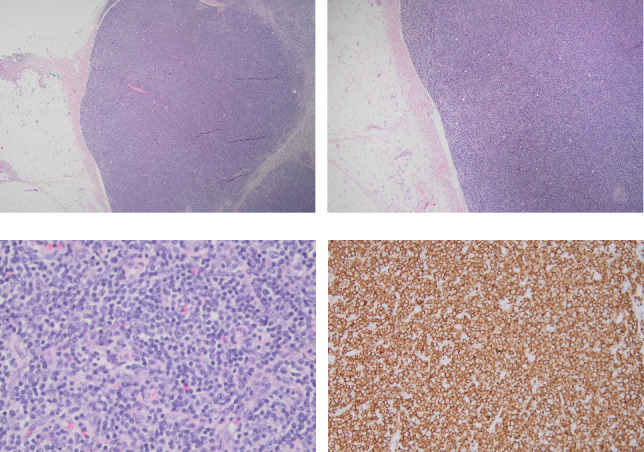
(a) (Hematoxylin and eosin, low-power, 2x magnification) demonstrates an expansile nodule of lymphoid tissue involving the subcutaneous adipose tissue; (b) (hematoxylin and eosin, low-power, 4x magnification) reveals a population of small uniform lymphocytes; (c) (hematoxylin and eosin, high-power, 40x magnification) notes a detailed view of a population of predominantly uniform small lymphocytes with occasional scattered larger cells with nucleoli (prolymphocytes); (d) immunohistochemical stain with strong positive staining for CD20.

An eighty-six-year-old female with a remote history of melanoma and basal cell carcinoma, longstanding history of rheumatoid factor and anti-CCP seropositive RA treated with infliximab for 20 years presented with a progressively enlarging subcutaneous mass of the left thigh. Laboratory evaluation noted a WBC count of 3.7 × 10^9^ (reference range 3.5–11.0 k/μL), hemoglobin 11.6 (12.0–16.0 g/dL), platelets 224 (140–450 k/μL), ESR 11 (0–30 mm/hr), and CRP < 3 (0–5 mg/dL). PET-CT noted a FDG avid subcutaneous mass on the left thigh with SUV of 5.6 without any accompanying lymphadenopathy or hepatosplenomegaly. Initial core biopsy demonstrated atypical lymphoid infiltrate concerning for low-grade B-cell lymphoma. Excisional biopsy of the 4.6-cm lesion showed a fairly well circumscribed subcutaneous nodule composed of sheets of small mature lymphocytes. On immunohistochemical stains, the lymphocytes were positive for CD20, CD43, and LEF1 and negative for Cyclin D1 and SOX11 with a Ki67 proliferation index of 25%–30% (Figures 1(a), 1(b), 1(c), and 1(d)). Flow cytometry demonstrated a monoclonal B-cell population negative for CD23 expression but positive for CD5, CD19, CD20, CD38, kappa light chain, and CD200 expression, overall consistent with chronic lymphocytic leukemia/small lymphocytic lymphoma (CLL/SLL). Given positive surgical margins, she underwent consolidative radiation at a dose of 2400 centiGrays in twelve fractions. Repeat WBC count was 5.4 × 10^9^ (reference range 3.5–11.0 k/μL) and LDH was 211 (reference range 100–235 U/L). Due to a RA flare precipitated by discontinuation of infliximab, abatacept was initiated with good control of symptoms.

## 3. Discussion

An increased risk of lymphoma in RA patients naïve to biological therapy has been reported, with aggressive, advanced stage non-GCB diffuse large B-cell lymphoma (DLBCL) being the most common subtype [5, 7]. B-cell clonal expansion in RA is theorized to be secondary to chronic immune stimulation, akin to the pathogenesis of Hodgkin's lymphoma or H. pylori, Sjogren's or Hashimoto's associated mucosa-associated lymphoma tissue (MALT) [5, 7]. Given this, lymphoma risk has been associated with increased RA disease activity and longer duration of RA [5]. Since immunosuppression is a risk factor for lymphoma, it has been hypothesized that lymphoma in RA may not only arise from increased RA disease activity but also from RA treatments themselves [8, 9]. For instance, withdrawal of methotrexate in RA patients has been associated with regression of EBV-associated lymphoproliferative disorder, suggesting that methotrexate may reactivate latent EBV [7]. Historically, there has been concern that TNF-alpha inhibitors in particular increase the risk of future malignancy. TNF-alpha inhibitors in combination with thiopurines have been associated with both B- and T-cell non-Hodgkin lymphomas when used in patients with inflammatory bowel disease (IBD) [10–12]. However, this association was not consistently present for IBD patients treated with TNF-alpha inhibitor monotherapy [10–12]. While many recent larger scale investigations of TNF-alpha inhibitors in RA specifically have not shown a consistent association, there may be an increased risk of nonmelanomatous skin cancer and non-Hodgkin lymphoma (particularly follicular lymphoma) in patients older than 65 years [5, 13–15].

CLL/SLL is an indolent B-cell lymphoma that is infrequently seen in RA. It comprises less than 10% of lymphoma in RA patients with an incident frequency lower than that of the general population [5]. CLL/SLL primarily involves the peripheral blood, bone marrow, and lymphatic system [1–3]. A diagnosis of CLL requires ≥ 5 x 10^9^/L monoclonal B lymphocytes in the peripheral blood, whereas SLL requires lymphadenopathy, splenomegaly, or extranodal disease with ≤ 5 × 10^9^/L monoclonal B lymphocytes in the peripheral blood [1, 3]. Flow cytometry with immunophenotyping demonstrates monoclonal B lymphocytes that coexpress CD5, CD19, CD20, and CD23 [16]. In equivocal cases, such as cases with CD23 negativity, additional immunophenotypic markers can distinguish between other lymphoproliferative disorders [16]. In this case, despite CD23 negativity, CD43, CD200, and LEF1 positivity support the diagnosis of CLL/SLL, whereas cyclin D1 and SOX11 negativity and LEF1 positivity exclude mantle cell and marginal zone lymphoma, respectively [16].

Extranodal and extramedullary manifestations are rare [2, 4]. Although the most common extranodal site of involvement is the skin, secondary skin cancer (both melanoma and nonmelanomatous), infectious and paraneoplastic cutaneous manifestations are more frequent than specific skin lesions (leukemic cutis), which occurs in only 4% of patients [2, 4, 6]. Leukemia cutis as the initial presentation of disease is uncommon as it typically manifests in the setting of advanced staged CLL/SLL, especially in patients older than 60 years [2, 6, 17]. Furthermore, isolated subcutaneous involvement is exceedingly rare, as leukemic cutis generally presents as visible erythematous plaques, papules, or nodules [6, 17].

Approximately 30%–50% of newly diagnosed CLL/SLL is classified as low risk, with only 8% of these patients requiring treatment in the first 5 years following diagnosis [3]. Treatment is indicated for patients with significant symptoms and complications from CLL/SLL, including B symptoms; progressive, bulky hepatosplenomegaly, and lymphadenopathy; progressive nonautoimmune cytopenias; steroid-refractory autoimmune cytopenias; and symptomatic extranodal disease threatening organ function [1, 3, 4]. While the current first line treatment regimens vary based on molecular and cytogenetic variables (ex. IGHV gene mutation and del(17p) TP53 gene mutations), they typically include targeted therapies such indefinite Bruton tyrosine kinase (BTK) inhibitors or fixed duration B-cell leukemia/lymphoma 2 (BCL2) inhibitors in combination with obinutuzumab (monoclonal anti-CD20 antibody) [1, 3].

In contrast, given the rarity of CLL/SLL-induced leukemia cutis, there is no consensus regarding treatment. Functional impairment from leukemia cutis or concomitant systemic disease are indications for standard targeted therapies, which often result in complete resolution of cutaneous lesions [2]. Careful observation is an option for isolated, minimally symptomatic leukemia cutis, as it has been reported to spontaneously resolve, particularly in early and localized disease [6, 17]. Other options, such as local radiation and UVB phototherapy, have been noted to achieve both partial and complete response [6, 17]. While a low Ki-67 index and a predominance of small B lymphocytes with minimal reactive inflammatory cells are associated with a favorable prognosis, leukemia cutis has an increased risk of Richter's transformation [4, 6]. Given the increased risk of both Richter's transformation and secondary skin cancers in CLL/SLL, regular surveillance is paramount [3, 4].

In conclusion, RA is infrequently associated with CLL/SLL. Isolated subcutaneous leukemia cutis as the initial manifestation of CLL/SLL is an exceedingly rare phenomenon. Localized, uncomplicated leukemia cutis may spontaneously resolve or respond to local radiation or phototherapy without the need for systemic targeted therapies. Given the association of RA with DLBCL and the increased risk of both secondary skin cancer and Richter's transformation in CLL/SLL, caution is warranted when considering TNF-alpha inhibitors in these patients.

## Data Availability

Data sharing is not applicable to this article as no new data were created or analyzed in this study.
